# Waist-to-height ratio is more closely associated with alanine aminotransferase levels than body mass index and waist circumference among population-based children: a cross-sectional study in Japan

**DOI:** 10.1186/s12887-015-0378-8

**Published:** 2015-05-17

**Authors:** Hirotaka Ochiai, Takako Shirasawa, Rimei Nishimura, Hinako Nanri, Tadahiro Ohtsu, Hiromi Hoshino, Naoko Tajima, Akatsuki Kokaze

**Affiliations:** Department of Public Health, Showa University School of Medicine, 1-5-8 Hatanodai, Shinagawa-ku, Tokyo, 142-8555 Japan; Division of Diabetes, Metabolism and Endocrinology, Department of Internal Medicine, Jikei University School of Medicine, Tokyo, Japan; Jikei University School of Medicine, Tokyo, Japan

**Keywords:** Waist-to-height ratio, Alanine aminotransferase, Schoolchildren, Population-based epidemiological study

## Abstract

**Background:**

An association between anthropometric measurements, including waist circumference (WC), and alanine aminotransferase (ALT) levels has been reported among adults. However, studies conducted among population-based elementary schoolchildren to date have been limited, especially in Japan, where the measurement of WC and blood collection are not usually performed in the annual health examination at elementary schools. The present study investigated the association between anthropometric measurements and ALT levels among population-based elementary schoolchildren in Japan.

**Methods:**

Subjects were fourth-grade schoolchildren (aged 9 or 10) from the town of Ina in Saitama Prefecture, Japan during 2004–2009. The height, weight, and WC of each subject were measured, and blood samples were drawn to measure ALT levels. Childhood overweight or obesity was defined according to the age- and sex-specific cut-off points proposed by the International Obesity Task Force. Spearman’s correlation coefficients between anthropometric measurements (body mass index (BMI), WC, and waist-to-height ratio (WHtR)) and ALT levels were calculated.

**Results:**

Data from 2499 subjects (1293 boys and 1206 girls) were analyzed. BMI, WC, and WHtR were significantly positively correlated with ALT levels; the correlation coefficient of ALT levels with WHtR was higher than that with BMI and WC in boys and girls. In the analysis stratified by physique (non-overweight/obesity, overweight, or obesity), all anthropometric measurements were significantly positively correlated with ALT levels among boys, while only WHtR was significantly positively correlated with ALT levels among girls. Moreover, the correlation coefficient of ALT levels with WHtR was more pronounced than that with BMI and WC in the non-overweight/obesity group, in the overweight group, and in the obesity group for each sex.

**Conclusions:**

The present study showed that WHtR was more closely associated with ALT levels than BMI and WC. Furthermore, only WHtR was significantly positively associated with ALT levels regardless of sex and physique. This study suggests that it is more useful to monitor WHtR than BMI and WC as a surrogate for ALT levels among population-based elementary schoolchildren.

## Background

Among the liver enzymes alanine aminotransferase (ALT), aspartate aminotransferase, and γ-glutamyltransferase, ALT is most closely related to liver fat accumulation [1]. Hepatic fat accumulation is reported to be strongly associated with insulin resistance, increased visceral fat, and hypoadiponectinemia [2]. Therefore, screening for ALT levels could help identify individuals at risk for these conditions. However, the measurement of ALT levels requires blood collection, which is invasive and could be impractical in large-scale, population-based epidemiological studies because of cost and subject burden.

In contrast, measurements of anthropometric variables such as height, weight, and waist circumference (WC) are non-invasive and easily conducted during health examinations. If anthropometric measurements showed a close correlation with ALT levels, they could be a useful surrogate for ALT levels among schoolchildren. Although the association between anthropometric measurements including WC and ALT has been reported among adults [3–6], studies conducted among population-based elementary schoolchildren to date have been limited, especially in Japan, where the measurement of WC and blood collection are not usually performed in the annual health examination at elementary schools.

The aim of the present study is to investigate the association between anthropometric measurements including WC and ALT among population-based elementary schoolchildren in Japan.

## Methods

The town of Ina in Saitama Prefecture has conducted a unique health-promotion program in addition to the annual national health checkups implemented according to the School Health Law of Japan. Studies regarding this program have been reported [7–9]. This study was conducted as part of the program.

### Subjects

Subjects were fourth-grade schoolchildren (aged 9 or 10) from elementary schools in the town of Ina during 2004–2009. Written informed consent was obtained from each subject’s parent or guardian. The study protocol was approved by the Medical Ethics Committee of Showa University School of Medicine.

Among 2526 subjects, 16 refused to participate in the program (participation rate: 99.4 %), and 11 were excluded because of incomplete data. Thus, data from 2499 subjects (1293 boys and 1206 girls) were analyzed.

### Data collection

Measurements of height, weight, and WC for each subject were made in the school’s infirmary or in a designated room to protect the subject’s privacy. For all measurements, subjects wore light clothing and were barefoot. Height was measured to the nearest 0.1 cm using a stadiometer, and body weight was measured to the nearest 0.1 kg using a scale. Body mass index (BMI) was calculated as weight (kg) divided by the square of height (m). Measurement of WC was performed in a standing position at the navel level while another examiner checked verticality from the side. The waist-to-height ratio (WHtR) was calculated as WC divided by height. Blood samples were drawn from the subjects to measure ALT levels. ALT was measured using an automated analyzer (H7600-020, Hitachi High-Technologies Co., Ltd., Tokyo, Japan). Data collection was conducted annually from 2004 to 2009. The same measurement protocol was undertaken annually throughout the study.

### Definition of overweight and obesity

Childhood overweight or obesity was defined according to the age- and sex-specific cut-off points proposed by the International Obesity Task Force [10].

### Data analysis

Medians and interquartile range (25th percentile-75th percentile) were used for continuous variables, and numbers (%) were used for categorical variables. The 95th percentile of ALT levels was calculated separately for boys and girls to define the cut-off limit of normality according to previous studies [11–13]. The cut-off limits were 28 IU/L for boys and 19 IU/L for girls in this study. The Wilcoxon rank-sum test or chi-square test was used to compare various characteristics between boys and girls and among each physique (non-overweight/obesity group vs. overweight group, overweight group vs. obesity group, non-overweight/obesity group vs. obesity group). Spearman’s correlation coefficients between anthropometric measurements (BMI, WC, and WHtR) and ALT levels were calculated. The correlation coefficient was applied for each sex. A *P* value <0.05 was considered statistically significant. All statistical analyses were performed using Statistical Analysis System software, version 9.3 (SAS Institute Inc., Cary, NC, USA).

## Results

Comparisons of characteristics between boys and girls are shown in Table 1. Boys were significantly heavier than girls. BMI and WHtR were significantly higher in boys than in girls. The proportion of overweight or obesity in boys was higher than that in girls. ALT levels were significantly higher in boys than in girls.

**Table 1:** Comparisons of characteristics between boys and girls

	Boys	Girls	
	(n = 1293)	(n = 1206)	*P* value^a^
Age (years)	9.0 (9.0–10.0)	9.0 (9.0–10.0)	0.532
Height (cm)	134.6 (130.7–138.7)	134.4 (130.5–139.0)	0.760
Weight (kg)	30.0 (27.0–34.3)	29.5 (26.3–33.8)	<0.001
Body mass index (kg/m^2^)	16.6 (15.4–18.4)	16.2 (15.1–17.8)	<0.001
Waist circumference (cm)	57.3 (54.0–62.3)	57.1 (54.0–61.1)	0.067
Waist-to-height ratio	0.43 (0.41–0.46)	0.43 (0.41–0.45)	0.035
Physique, n (%)			
Non-overweight/obesity	1082 (83.7)	1059 (87.8)	0.011
Overweight^b^	168 (13.0)	121 (10.0)	
Obesity^b^	43 (3.3)	26 (2.2)	
ALT (IU/L)	13.0 (11.0–17.0)	12.0 (10.0–14.0)	<0.001

*ALT* alanine aminotransferase, *IQR* interquartile range

Except where n (%) is indicated, values are median (IQR: 25th percentile-75th percentile)

^a^Wilcoxon rank-sum test or chi-square test

^b^Overweight and obesity were defined by the criteria of the International Obesity Task Force

Spearman’s correlation coefficients of anthropometric measurements with ALT were calculated for each sex (Table 2). All anthropometric measurements (BMI, WC, and WHtR) were significantly positively correlated with ALT in boys; the correlation coefficients of BMI, WC, and WHtR with ALT were 0.353 (*P* <0.001), 0.340 (*P* <0.001), and 0.365 (*P* <0.001), respectively. Furthermore, the correlation coefficient of ALT with WHtR was higher than that with BMI and WC. Similar results were obtained in girls; the correlation coefficients of BMI, WC, and WHtR with ALT were 0.235 (*P* <0.001), 0.210 (*P* <0.001), and 0.250 (*P* <0.001), respectively.

**Table 2:** Correlations of anthropometric measurements with alanine aminotransferase levels by sex

Anthropometric measurements	Boys (n = 1293)		Girls (n = 1206)	
	r^a^	*P* value	r^a^	*P* value
Body mass index	0.353	<0.001	0.235	<0.001
Waist circumference	0.340	<0.001	0.210	<0.001
Waist-to-height ratio	0.365	<0.001	0.250	<0.001

^a^Values are Spearman’s correlation coefficients

Table 3 shows comparisons of anthropometric measurements and ALT levels among each physique by sex. In both sexes, anthropometric measurements were significantly higher in the obesity group than in the overweight group and significantly higher in the overweight group than in the non-overweight/obesity group. ALT levels in the obesity group were significantly higher than those in the overweight group or in the non-overweight/obesity group regardless of sex. The proportion of boys with ALT above the normal range (ALT >28 IU/L) was 46.5 % in the obesity group, 13.7 % in the overweight group, and 1.8 % in the non-overweight/obesity group; the proportion of girls with ALT above the normal range (ALT >19 IU/L) was 34.6 % in the obesity group, 9.1 % in the overweight group, and 3.3 % in the non-overweight/obesity group.

**Table 3:** Comparisons of anthropometric measurements and alanine aminotransferase levels among each physique by sex

Anthropometric measurements	Non-overweight/obesity	Overweight	Obesity
Boys (n = 1293)	(n = 1082)	(n = 168)	(n = 43)
Body mass index (kg/m^2^)	16.2 (15.2–17.3)	20.6 (19.8–21.5) ^a^	24.9 (23.8–26.7) ^a, b^
Waist circumference (cm)	56.3 (53.5–59.3)	69.4 (66.0–73.0) ^a^	^a^ 83.0 (77.1–85.9) ^a, b^
Waist-to-height ratio	0.42 (0.40–0.44)	0.50 (0.49–0.53) ^a^	0.59 (0.57–0.61) ^a, b^
ALT (IU/L)	13.0 (11.0–16.0)	16.5 (11.0–24.0) ^a^	26.0 (16.0–41.0) ^a, b^
ALT above the normal range^c^, n (%)	19 (1.8)	23 (13.7) ^a^	20 (46.5) ^a, b^
Girls (n = 1206)	(n = 1059)	(n = 121)	(n = 26)
Body mass index (kg/m^2^)	15.9 (15.0–17.1)	20.6 (19.9–21.4) ^a^	25.0 (23.3–26.5) ^a, b^
Waist circumference (cm)	56.3 (53.5–59.3)	69.6 (65.9–72.3) ^a^	80.5 (77.8–82.8) ^a, b^
Waist-to-height ratio	0.42 (0.40–0.44)	0.50 (0.48–0.53) ^a^	0.57 (0.54–0.60) ^a, b^
ALT (IU/L)	12.0 (10.0–14.0)	14.0 (12.0–16.0) ^a^	18.0 (13.0–26.0) ^a, b^
ALT above the normal range^c^, n (%)	35 (3.3)	11 (9.1) ^b^	9 (34.6) ^b, c^

*ALT* alanine aminotransferase, *IQR* interquartile range

Except where n (%) is indicated, values are median (IQR: 25th percentile-75th percentile)

^a^ *P* <0.05 compared with non-overweight/obesity group by Wilcoxon rank-sum test or chi-square test

^b^ *P* <0.05 compared with overweight group by Wilcoxon rank-sum test or chi-square test

^c^ALT >28 IU/L for boys and >19 IU/L for girls

Next, Spearman’s correlation coefficients of anthropometric measurements with ALT levels among each physique were calculated by sex (Table 4). In boys, all anthropometric measurements were significantly positively correlated with ALT levels and the correlation coefficient of ALT levels with WHtR was higher than that with BMI and WC. The correlation coefficient between WHtR and ALT levels was 0.615 (*P* <0.001) in the obesity group, 0.256 (*P* <0.001) in the overweight group, and 0.227 (*P* <0.001) in the non-overweight/obesity group. In the analysis of girls, the correlation coefficient of ALT was statistically significant only for WHtR regardless of physique. The correlation coefficient of WHtR with ALT levels was 0.448 (*P* = 0.022) in the obesity group, 0.257 (*P* = 0.005) in the overweight group, and 0.154 (*P* <0.001) in the non-overweight/obesity group.

**Table 4:** Correlations of anthropometric measurements with alanine aminotransferase levels among each physique by sex

Anthropometric measurements	Non-overweight/obesity		Overweight		Obesity	
	r^a^	*P* value	r^a^	*P* value	r^a^	*P* value
Boys (n = 1293)	(n = 1082)		(n = 168)		(n = 43)	
Body mass index	0.209	<0.001	0.2555	<0.001	0.543	<0.001
Waist circumference	0.192	<0.001	0.219	0.004	0.479	0.001
Waist-to-height ratio	0.227	<0.001	0.2557	<0.001	0.615	<0.001
Girls (n = 1206)	(n = 1059)		(n = 121)		(n = 26)	
Body mass index	0.138	<0.001	0.123	0.177	0.346	0.083
Waist circumference	0.104	<0.001	0.056	0.539	0.320	0.111
Waist-to-height ratio	0.154	<0.001	0.257	0.005	0.448	0.022

^a^Values are Spearman’s correlation coefficients

## Discussion

In the present study, anthropometric measurements were positively correlated with ALT levels. Furthermore, the correlation coefficient of ALT levels was higher with WHtR than with BMI and WC, and only WHtR was significantly positively correlated with ALT levels regardless of sex and physique. To the best of our knowledge, this is the first study regarding the association between anthropometric measurements including WC and ALT levels among population-based elementary schoolchildren in Japan, where measurement of WC and blood collection are not commonly performed in the annual health examination at elementary schools. However, the study results must be interpreted carefully.

In this study, characteristics were significantly different between boys and girls: some anthropometric variables and ALT levels were significantly higher in boys than in girls. A recent study showed that sex differences in body composition are present very early in life [14]. Furthermore, ALT levels were reported to be higher in males than in females among children and adolescents [15–17]. Therefore, we analyzed data separately by sex to examine the association between anthropometric measurements and ALT levels.

Anthropometric measurements (BMI, WC, and WHtR) were positively correlated with ALT levels and the correlation coefficient of ALT levels was higher with WHtR than with BMI and WC. Some studies showed that BMI and WC were correlated with ALT levels [15, 17, 18]. In addition, previous studies have reported that ALT concentrations have a strong association with visceral fat accumulation [5], while the correlation of intra-abdominal fat with WHtR was higher than that with BMI and WC [19]. These findings are not inconsistent with our results.

ALT levels were higher in the obesity group than in the overweight group and higher in the overweight group than in the non-overweight/obesity group. A recent study showed that ALT levels were strongly associated with BMI and increased progressively from the lowest to the highest strata of BMI [20]. In fact, ALT levels were reported to be higher in obese children than in non-obese children [17]. Furthermore, a previous study showed that obese children were significantly more likely to have higher levels of ALT than were overweight or normal-weight children [21]. These results were consistent with our study results.

Sex differences of the associations between anthropometric measurements and ALT levels were observed; the correlation coefficients between anthropometric measurements and ALT in boys were higher than those in girls, and the propensity was similar in the stratified analysis by each physique. A recent study showed that the correlation coefficient of anthropometric variables with ALT levels was higher in boys than in girls [17], while some studies have reported the impact of sex on ALT levels [22, 23]. One reason for the presence of sex differences could be due to sex hormones. A recent study suggested that sex hormones affect the distribution of fat, and males are more likely to distribute excess body fat in the intra-abdominal compartment [24]. Visceral fat accumulation has shown a strong correlation with liver steatosis [25]. Moreover, the severity of fatty liver was positively related to anthropometric measurements including BMI and WC [26], whereas serum ALT levels increased with the degree of steatosis [27]. Therefore, sex hormones could partially explain the sex differences in the relationship between anthropometric variables and ALT levels. However, the influence of sex hormones on the present study results was not likely to be substantial because all subjects in this study were prepubertal. Another reason might be due to liver volume. Recent studies showed that serum ALT levels were correlated with liver fat [28, 29], while mean liver attenuation, which is measured from computed tomography scans and is a validated measure of liver fat, varied significantly by sex [30]. Because information on sex hormones and liver fat was not collected in this study, further studies will be needed to elucidate the sex differences of the association between anthropometric measurements and ALT levels.

In the present study, correlation coefficients of anthropometric measurements with ALT levels were calculated among each physique in both sexes. As a result, only WHtR was significantly positively correlated with ALT levels regardless of sex and physique. Additionally, the correlation coefficient of WHtR with ALT levels was higher in the obese group than in the overweight group and in the non-overweight/obese group for each sex. These results suggest that WHtR is a more effective tool for predicting ALT levels than BMI and WC, especially in obese children, because the association between WHtR and ALT was strongest in the obese group.

The strength of the present study is that measurements of all anthropometric variables (height, weight, and WC) and ALT were conducted among more than 2000 population-based elementary schoolchildren. However, there are some potential limitations to this study. First, the present study was based on a cross-sectional design. Therefore, a causal relationship between anthropometric measurements and ALT levels was not determined. Second, the correlation coefficients between anthropometric measurements and ALT levels in the obesity group were based on a relatively small sample size compared with the overweight group and the non-overweight/obesity group, especially among girls. Therefore, the results need to be verified in a larger population. Third, subjects in our study were from one town in Japan. Thus, it might be difficult to generalize these results to other populations. Finally, although the relationship between anthropometric variables and ALT levels was analyzed separately for sex because sex was reported to be associated with ALT levels in other studies [22, 23], the possibility of residual confounding exists. For example, information regarding alcohol intake, hepatitis B virus (HBV) infection, and hepatitis C virus (HCV) infection, which are known risk factors for elevated ALT levels [31, 32], was not obtained in our study. However, the law prohibits minors under 20 years of age from drinking alcohol in Japan [33]. In addition, Tanaka et al. reported that the prevalence of HBV among schoolchildren was less than 0.05 %, while that of HCV among schoolchildren was less than 0.03 % [34]. Thus, alcohol intake, HBV, and HCV infections were not likely to have a substantial impact on the present study results.

## Conclusions

The present study showed that WHtR was more closely associated with ALT levels than BMI and WC. Furthermore, only WHtR was significantly positively associated with ALT levels regardless of sex and physique. This study suggests that it is more useful to monitor WHtR than BMI and WC as a surrogate for ALT levels among population-based elementary schoolchildren, especially obese children.
